# Airway management in patients with surgical treatment of oral cavity carcinoma

**DOI:** 10.1186/s12871-025-03048-4

**Published:** 2025-04-23

**Authors:** Patrick Sturm, Holger Gaessler, Christel Weiß, Alexander Schramm, Frank Wilde, Marcel Ebeling, Andreas Sakkas

**Affiliations:** 1 Department of Oral and Maxillofacial Surgery, University Hospital Ulm, Ulm, Germany; 2 Department of Oral and Plastic Maxillofacial Surgery, Federal Armed Forces Hospital Ulm, Oberer Eselsberg 40, 89081 Ulm, Germany; 3 Department of Anaesthesiology, Intensive Care Medicine, Emergency Medicine and Pain Therapy, Federal Armed Forces Hospital Ulm, Ulm, Germany; 4 Medical Statistics and Biomathematics, University Medical Centre Mannheim, Heidelberg University, Mannheim, Germany

**Keywords:** Difficult airway, Videolaryngoscopy, Tracheostomy, Airway management, Oral cavity carcinoma

## Abstract

**Background:**

Local tumor-related anatomical changes can complicate the anesthetic airway management of patients with carcinoma of the oral cavity. The aim of this study was to investigate whether there are predictive factors for the occurrence of a difficult airway in this patient cohort and whether a difficult airway influences postoperative outcome. In addition, the influence of an intraoperative tracheostomy on postoperative outcome was to be analyzed.

**Methods:**

The treatment records of 201 patients with oral cavity carcinoma who underwent surgery between 2012 and 2023 in a single center were retrospectively analyzed. The definition of difficult airway corresponded to the current S1 guideline of the German Society of Anesthesiology and Intensive Care Medicine from 2015. An association between possible predictive factors and a difficult airway was investigated. The influence of BMI, Mallampati score and Cormack/Lehane score on the number of intubation attempts was analyzed separately. Furthermore, the influence of a difficult airway on the duration of intubation and the duration of the postoperative inpatient stay as well as the postoperative ICU stay was investigated. In addition, the association between an intraoperative tracheostomy and the duration of intubation as well as the duration of the postoperative inpatient stay was analyzed.

**Results:**

Difficult airway occurred in 15 patients (7,5%) and 136 (68%) underwent intraoperative tracheostomy. An indirect laryngoscopy was used in advance in 32,8% of the total patients and 45,4% of the patients undergoing revision surgery. Among the investigated variables, no predictive factors for a difficult airway could be identified. Regarding the number of intubation attempts required, a higher BMI and Mallampati score did not lead to increased number of intubation attempts; however, patients with a Cormack/Lehane score of 3 were significantly more likely to require 2 attempts than patients with a score of 1 or 2 (*p* = 0.0225). The success rate of first intubation attempt was 78% with videolaryngoscopy, compared to 95,5% when direct laryngoscopy was used (*p* = 0,0008). A difficult airway did not lead to prolonged postoperative ICU stay and total hospitalisation length. Patients with an intraoperative tracheostomy had a significantly longer mechanical ventilation and total hospitalisation length than patients without (*p* < 0.0001).

**Conclusion:**

Within the limitations of this study, no patient-specific predictors for a difficult airway were identified in patients with oral cavity carcinoma. Videolaryngoscopy in advance did not increase the success rate of the first intubation attempt compared to direct laryngoscopy. Despite this, videolaryngoscopy may be a preferable approach in this population, especially in patients undergoing revision surgeries. The results highlight the importance of a careful preoperative assessment with clearly defined criteria for a difficult airway and appropriate anaesthesiological preparation to avoid complications during intubation.

**Trial registration:**

Ethics committee of the University of Ulm, approval reference: 115/23, approval date: 08.05.2023.

## Introduction

The incidence of oral cavity cancer in Germany in 2020 was estimated at ninth place (3,5%) for men and fourteenth place (1,8%) for women respectively [1]. Alcohol and tobacco consumption are the main risk factors responsible for this development, an infection with the human papillomavirus, especially type 16 and 18, represents a further risk factor [2–5].

Airway management in patients with oral cavity carcinoma can be complicated by the influence of many anatomical deformities [6]. Depending on its size, growth and localization, oral cavity carcinoma can lead to obstruction of the upper airway, increasing the risk of a difficult airway management [6]. Also, patients with previous radiotherapy in the head and neck region could present a difficult airway through the soft tissue restriction [7]. A restricted mouth opening and head reclination could also have a major impact on airway management [8, 9]. Furthermore, obesity (body mass index (BMI) ≥ 30) has been described to increase the risk of difficult mask ventilation and difficult intubation [10–12]. An increased Mallampati score appears also to be a reliable predictor for a difficult laryngoscopy [10]. Further risk factors for a difficult airway like a high Mallampati and Cormack/Lehane Score, advanced age, high ASA-Score, male gender and revision surgery have already been reported in the international literature [13–16].

A conscientious anaesthesiological airway management combined with appropriate surgical therapy can shorten the postoperative intensive care unit stay and total hospitalisation length, and thus ensure a better patient’s outcome, promoting the quality of life and rehabilitation. Consequently, an effective reduction in the economic burden on the healthcare system can be achieved [17–19].

Intraoperative tracheostomy is often used electively in major surgery for oral cavity tumours with neck dissection and/or extended flap reconstructions to prevent the risk of postoperative upper airway obstruction, due to the surgically induced anatomical soft-tissue changes [20, 21]. However, tracheostomy may be associated with complications such as bleeding, infection, impaired wound healing, pneumothorax, mispositioning of the tracheostoma, perforation of the trachea, tracheal stenosis or tracheomalacia, some of which may be potentially life-threatening [22]. Depending on the postoperative course regarding intraoral and cervical swelling, it has been shown that patients with tracheostomy appear to have a significantly longer postoperative hospital stay than patients without [21–24].

The primary aim of this study was to assess the incidence of difficult airway in patients with surgical treatment of oral cavity carcinoma and determine whether there are patient-specific predictive factors for a difficult airway in this patient cohort. The secondary aim was to investigate whether a difficult airway and an intraoperative tracheostomy has an impact on postoperative outcome in terms of length of postoperative ICU stay and total hospitalisation length. To the best of our knowledge, this is the first study investigating the airway management in patients with oral cavity cancer regarding the national applied definition for difficult airway.

## Methods

### Patient collection

For this observational retrospective single-center study, we reviewed the medical records of all patients with oral cavity carcinoma who were surgically treated under general anaesthesia in our department of oral and plastic maxillofacial surgery between July 2012 and February 2023. Records were retrieved from our hospital electronic database.

Ethical approval for this study (approval reference: 115/23, approval date: 08.05.2023) was obtained from the ethics committee of the University of Ulm, Germany, and the study was performed in accordance with the Declaration of Helsinki 1964 and its later amendments (World Medical Association, Declaration of Helsinki).

We enrolled patients of all ages with surgical treatment of oral cavity carcinomas of all entities. Exclusion criteria were (1) patients with a preoperatively existing tracheostoma and (2) incomplete medical charts.

### Patient screening

Our clinic’s standard procedure for patients undergoing surgical treatment for oral cavity carcinoma includes a premedication consultation by a board-certified anaesthesiologist. According to the type and extent of surgical intervention planned, an interdisciplinary decision is made preoperatively to whether a tracheostomy should be performed preoperatively to secure the airway. The type of laryngoscopy (direct/fibreoptic/videolaryngoscopy) to be performed is selected in advance by the attending anaesthesiologist. Considering the extent of surgical intervention, especially in patients with combined neck dissection and free flap defect reconstruction, the clinic protocol is to transfer these patients to the ICU postoperatively and extubate only on the first postoperative day, after interdisciplinary assessment. After successful weaning and extubation, the patient is usually transferred to the surgical ward on the same day.

### Difficult airway

The S1 guideline of the German Society of Anaesthesiology and Intensive Care Medicine (DGAI) from 2015 was applied in this study. Accordingly, a difficult airway is defined as problems that can occur during airway management under board-certificated specialist standards and is present if one of the following sub-definitions is met [25]:

- Ventilation using the face mask or an extraglottic airway adjunct is defined as difficult or impossible if ventilation is inadequate or even fails completely due to one or more problems: leakage, massive leakage and resistance during inspiration or expiration. The placement of an extraglottic airway adjunct is described as difficult if several placement attempts are necessary.
- Difficult laryngoscopy is defined as the inability to visualize the glottis using direct laryngoscopy. This corresponds to a Cormack/Lehane score 3 or 4 laryngoscopy finding.
- Difficult endotracheal intubation is present when multiple intubation attempts are necessary.

These definitions are similar in principle to the Difficult Airway Society 2015 guidelines used in the UK [26]. We defined “intubation duration” as the duration of intubation for non-tracheotomised patients and as the duration of mechanical ventilation for tracheotomized patients for the purposes of further analysis. Similarly, the end of the intubation period was defined as the time point of extubation for non-tracheotomised patients and the time point of weaning from mechanical ventilation for tracheotomized patients.

### Data collection

Data were collected from patients’ electronic hospital charts and patients were anonymized before data analysis. Extracted data comprised patient’s age, gender, BMI, smoking and alcoholic status, ASA score, pre-irradiation, performance of an intraoperative tracheostomy, tumour entity, stage and localisation, Mallampati score, Cormack/Lehane score, mouth opening, head reclination, type of surgical intervention, airway management (difficult/regular), mask ventilation (difficult/regular), ventilation and laryngoscopy, number of intubation attempts, and the postoperative outcome (intubation/mechanical ventilation duration, length of postoperative ICU stay, length of total hospitalisation).

Cormack/Lehane score could not be ascertained if laryngoscopy was not performed directly, but rather with fibreoptic or videolaryngoscopy. Mouth opening was defined as “restricted” by an opening of 3 cm. Cases with non-reported tumour stage, Mallampati and Cormack/Lehane score, and mask ventilation modality were documented as “not applicable”. If individual details regarding mouth opening or head reclination were not reported, a physiological condition was assumed.

### Statistical analysis

Data were centralized in an electronic format using Microsoft Excel software and analyzed descriptively. Statistical analysis was performed using SAS®, Release 9.4 software (SAS Institute Inc., Cary, NC, USA). Descriptive statistics were used to describe baseline patient characteristics. All categorical variables were expressed as absolute values (n) and relative incidences (%). For metric variables, the standard deviation was calculated. A multivariable analysis was performed to find associations between the possible influencing variables and a difficult airway. Continuous and within the sample normally distributed and connected variables (e.g. patient’s age and BMI) were analysed with the t-test for two connected samples. In case of continuous variables (association between difficult airway and tracheostomy on length of intubation, length of ICU stay and total hospitalization length), which were connected within the sample but not normally distributed, the contingency table was analysed with the Wilcoxon two-sample test for two connected samples. The Kruskal-Wallis test (BMI and number of intubation attempts) was used to compare more than two unconnected samples with regard to a quantitative, non-normally distributed variable. The exact Cochran-Armitage trend test was used to check whether two groups differed regarding an ordinally scaled characteristic (association between ASA-grade, Mallampati score and difficult airway). To check the association between two nominally scaled characteristics, the chi-square test was used for analysis (association between gender and tumour with a difficult airway. If the requirements of the chi-square test were not met, the Fisher exact test was used instead. Spearman’s correlation coefficient was used to examine the relationship between two ordinally scaled or two quantitative characteristics (association between Mallampati and Cormack/ Lehane score and number of intubation attempts). The Kolmogorov-Smirnov test showed a normal data distribution for the variables patient’s age (*p* = 0,308), BMI (*p* = 0,364), and total hospitalization length (*p* = 0,207), while length of intubation and length of ICU stay were not normally distributed (*p* < 0,001). A two-sided *p* value of less than 0.05 was considered statistically significant.

## Results

### Demographic distribution

A total of 201 patients were included in the analysis. There were more males (117/201; 58%) than females (84/201; 42%) and the male: female ratio was 1,39:1. The patient’s age at the time of surgery ranged from 27 to 88 years, with a mean ± SD age was 64,19 ± 11,20 years. Most patients (90%) were older than 50 years. The mean ± SD BMI at the time of admission was 25,72 ± 5,22. Baseline demographics, clinical and anaesthesiological findings and outcomes of the overall study population are presented in Table 1.

**Table 1:** Baseline demographics, clinical and anaesthesiological findings and outcomes of the overall study population

	Study Population	
	n	%
Total	201	100%
Gender		
male	117	58%
female	84	42%
Age		
< 30 years	2	1%
30-<50 years	18	9%
≥ 50 years	181	90%
BMI		
< 25	99	49%
25-<30	67	33%
≥ 30	35	18%
Smoking Status		
positive	119	59%
negative	82	41%
Alcohol Status		
positive	96	48%
negative	105	52%
Tumour Localisation		
anterior mouth floor left	18	9,0%
anterior mouth floor middle	22	10,9%
anterior mouth floor right	19	9,5%
hard palate middle	1	0,5%
hard palate right	2	1,0%
maxillary alveolar process left	2	1,0%
maxillary alveolar process right	8	4,0%
buccal planum left	10	5,0%
buccal planum right	14	7,0%
lateral mouth floor left	2	1,0%
lateral mouth floor right	3	1,5%
mandibular alveolar process left	23	11,4%
mandibular alveolar process middle	1	0,5%
mandibular alveolar process right	13	6,5%
lateral tongue left	39	19,3%
lateral tongue right	23	11,4%
tongue tip	1	0,5%
Tumour entity		
squamous cell carcinoma	197	98%
adenoid cystic carcinoma	2	1%
polymorphic adenocarcinoma	1	0,5%
basal cell adenocarcinoma	1	0,5%
Tumour stage		
Tis	5	3%
T1	86	43%
T2	61	30%
T3	35	17%
T4	5	3%
not applicable*	9	4%
Previous irradiation		
yes	10	5%
no	191	95%
Tracheostomy		
yes	136	68%
no	65	32%
Revision surgery		
yes	22	11%
no	179	89%
ASA score		
1	0	0%
2	51	25,5%
3	149	74%
4	1	0,5%
5	0	0%
Mallampati score		
1	60	30%
2	98	49%
3	29	14%
4	12	6%
not applicable*	2	1%
Mouth opening		
physiological	24	12%
restricted (≤ 3cm)	177	88%
Head reclination		
physiological	42	21%
restricted	159	79%
Mask ventilation		
difficult	0	0%
regular	198	98,5%
not applicable*	3	1,5%
Cormack/Lehane score		
1	111	55%
2	21	10,5%
3	2	1%
4	0	0%
not applicable*	67	33,5%
Ventilation method		
nasotracheal	104	51,7%
orotracheal	96	47,8%
tracheal	1	0,5%
Laryngoscopy method		
direct laryngoscopy	135	67,2%
videolaryngoscopy	41	20,4%
fibreoptic	24	11,9%
awake tracheostomy in local anaesthesia	1	0,5%
Intubation attempts		
1	186	92,5%
2	13	6,5%
3	2	1%
Difficult airway		
yes	15	7,5%
no	186	92,5%

Abbreviations: *n* = number; %=percentage; BMI = body mass index; ASA = American Society of Anaesthesiology

* Unavailable data

On average, patients were intubated for a duration of 0,80 ± 1,00 days, with a minimum of zero (intubation only on the day of surgery) and a maximum of 13 days respectively. The average length of postoperative stay in the ICU was 0,84 ± 1,21 days, ranged from zero (no stay in the ICU) and 16 days. The average length of the total hospitalisation (including stay in the ICU) was 12,51 ± 6,73 days, ranged from one to 35 days.

### Difficult airway

**Fig. 1:**
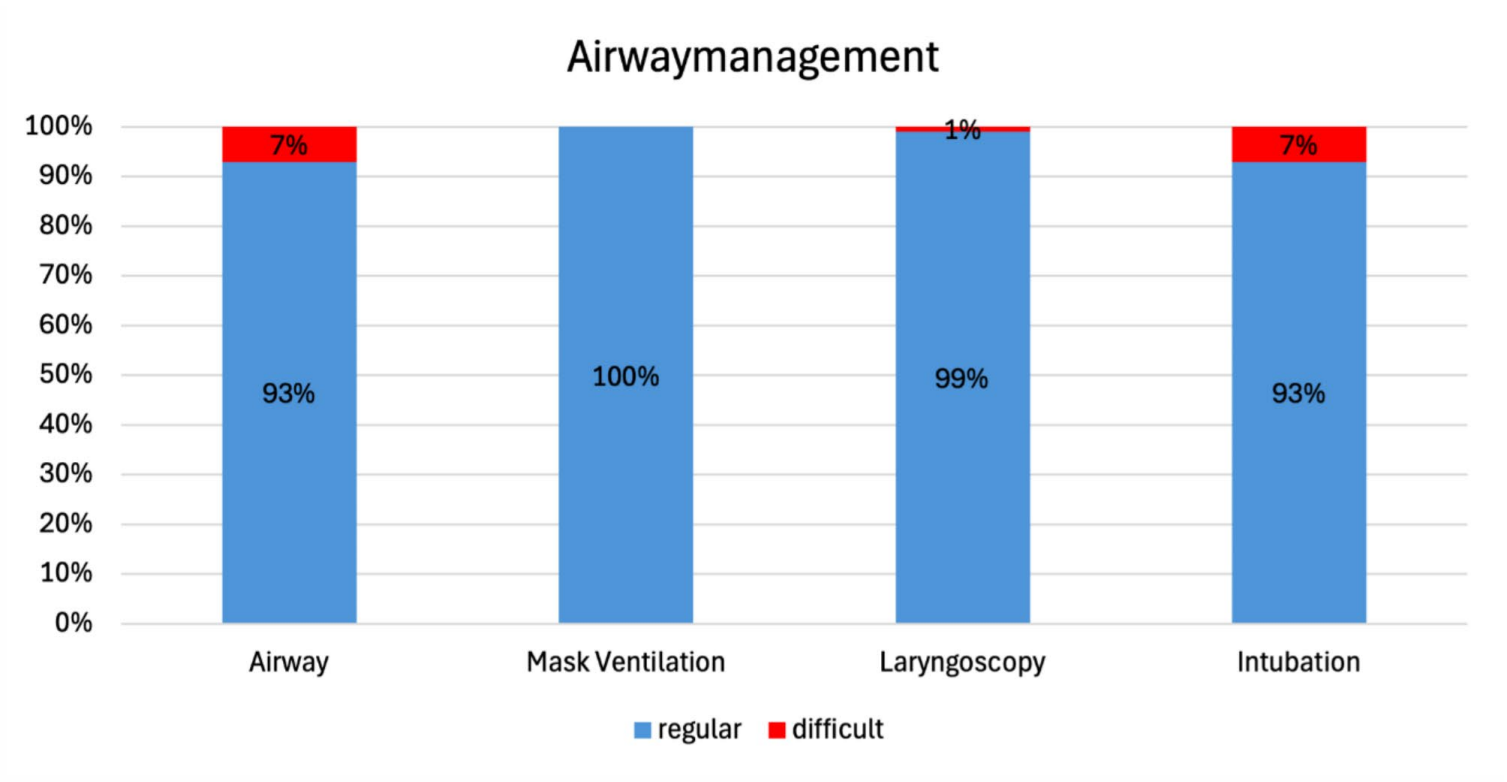
Airway modality regarding ventilation, laryngoscopy and intubation according to the study`s definition of a difficult airway

15 (7,5%) of the 201 patients had a difficult airway. A difficult mask ventilation was not reported in any patient, while a difficult intubation was documented in 15 (7,5%) patients. Of these 15 patients, two had also a difficult laryngoscopy. Accordingly, among the 201 patients, 13 (6,5%) had a difficult airway due a difficult intubation and two (1%) due both difficult laryngoscopy and difficult intubation (Fig. 1). Among the 15 patients with difficult airway, 6 (40%) were intubated using direct laryngoscopy and 9 (60%) using videolaryngoscopy. Difficult intubation in patients using videolaryngoscopy was with a rate of 21,9% (*n* = 9/41) significantly more frequent than in those using direct laryngoscopy with a rate of 4,4% (*n* = 6/135) (*p* = 0,0015, Fisher`s exact test) (Table 2). Considering demographic data of the patients with difficult airway, 53,3% (*n* = 8/15) had a positive alcohol anamnesis, 66,6% (*n* = 10/15) were smokers, 94% (*n* = 14/15) had an ASA score 2–3, and 60% (*n* = 9/15) had a tumour T stage 2–3.

**Table 2:** Association between laryngoscopy method and difficult airway

Laryngoscopy method	Regular airway (n)	Difficult airway (n)	Number of patients (n)	p-Value
Direct laryngoscopy	129	6	135	
Videolaryngoscopy	32	9	41	0,0015*
fibreoptic	24	0	24	
Awake tracheostomy in local anaesthesia	1	0	1	
Alternative laryngoscopy	66	9	57	0,0411**

Abbreviations: *n* = number; alternative laryngoscopy = videolaryngoscopy + fibreoptic + awake tracheostomy in local anaesthesia; significance level = 0.05

* Fisher`s exact test, ** Fisher`s exact test

### Multivariable analysis

The multivariable analysis revealed no significant correlation between gender, patient’s age, BMI, tumour localisation, tumour stage, previous irradiation, revision surgery, ASA score, Mallampati score, mouth opening and head reclination and the incidence of a difficult airway (Table 3). Of the 22 patients who underwent revision surgery, 12 patients were intubated using direct laryngoscopy, 9 patients using a videolaryngoscope and one patient received an awake tracheostomy under local anaesthesia.

**Table 3:** Association between patient-specific variables and incidence of difficult airway

	Difficult airway				*p*-Value
	yes		no		
	n	%	n	%	
Gender					
male	11	5,5%	106	52,7%	0,2170*
female	4	2,0%	80	39,8%	
Age					
< 30 years	0	0%	2	1,0%	0,3729**
30-<50 years	1	0,5%	17	8,5%	
≥ 50 years	14	7,0%	167	83,0%	
BMI					
< 25	7	3,5%	92	45,7%	0,4750**
25-<30	7	3,5%	60	29,9%	
≥ 30	1	0,5%	34	16,9%	
Tumour localisation					
anterior mouth floor	5	2,5%	54	26,9%	
hard palate middle	0	0,0%	3	1,5%	
maxillary alveolar process	2	1,0%	8	4,0%	
buccal planum	2	1,0%	22	10,9%	0,608***
lateral mouth floor	0	0,0%	5	2,5%	
mandibular alveolar process	3	1,5%	34	16,9%	
tongue	3	1,5%	60	29,8%	
Tumour stage					
Tis	1	0,5%	4	2,0%	0,2593*
T1	5	2,5%	81	40,3%	
T2	3	1,5%	58	28,8%	
T3	5	2,5%	30	14,9%	
T4	1	0,5%	4	2,0%	
not applicable*****	0	0,0%	9	4,5%	
Previous irradiation					
yes	0	0,0%	10	5,0%	1,0000***
no	15	7,5%	176	87,5%	
Revision surgery					
yes	0	0,0%	22	10,9%	0,3807***
no	15	7,5%	164	81,6%	
ASA score					
1	0	0,0%	0	0,0%	0,5442****
2	6	3,0%	45	22,4%	
3	8	4,0%	141	70,1%	
4	1	0,5%	0	0,0%	
5	0	0,0%	0	0,0%	
Mallampati score					
1	3	1,5%	57	28,3%	1,0000****
2	11	5,5%	87	43,3%	
3	0	0,0%	29	14,4%	
4	1	0,5%	11	5,5%	
not applicable*****	0	0,0%	2	1,0%	
Mouth opening					
physiological	13	6,5%	164	81,6%	0,6957***
restricted	2	1,0%	22	10,9%	
Head reclination					
physiological	11	5,5%	148	73,6%	0,5220***
restricted	4	2,0%	38	18,9%	

Abbreviations: *n* = number; %=percentage; BMI = body mass index; ASA = American Society of Anaesthesiology; significance level = 0.05

* chi-square test; ** t-test; *** Fisher`s exact test; **** exact Cochrane-Armitage trend test; ***** unavailable data

In 134 out of 201 patients, a Cormack/Lehane score was regularly documented after direct laryngoscopy, while in 67 patients not (66 with indirect laryngoscopy and one with incomplete data). 92,5% of the patients (*n* = 186/201) required one intubation attempt, 6,4% (*n* = 13/201) two intubation attempts and 1,1% (*n* = 2/201) three intubation attempts. An increased BMI or Mallampati score did not lead to increased number of intubation attempts (Table 4). Considering the Cormack/Lehane score, a score of 3 was significantly associated with two intubation attempts (*p* = 0.0225, exact Cochran-Armitage trend test). Considering the laryngoscopy method, 9 out of 41 patients (21,9%) with videolaryngoscopy and 6 out of 135 patients (4,4%) with direct laryngoscopy needed more than one intubation attempt, stating that the success rate of first intubation attempt was 78% (*n* = 32/41) when videolaryngoscopy was used, compared to 95,5% (*n* = 129/135) when direct laryngoscopy was used (*p* = 0,0008, Fisher`s exact test). The association between BMI, Mallampati, Cormack/Lehane score, and laryngoscopy method with the number of intubation attempts is presented in Table 4.

**Table 4:** Association between BMI, Mallampati, Cormack/Lehane score, and laryngoscopy method with the number of intubation attempts

	Intubation attempts						*p*-Value
	1 attempt		2 attempts		3 attempts		
	n	%	n	%	n	%	
**BMI**							
< 25	92	45,7%	7	3,5%	0	0,0%	0,3602*
25 - <30	60	29,9%	5	2,5%	2	1,0%	
≥ 30	34	16,9%	1	0,5%	0	0,0%	
**Mallampati Score**							
1	57	28,3%	3	1,5%	0	0,0%	0,9640**
2	87	43,3%	9	4,5%	2	1,0%	
3	29	14,4%	0	0,0%	0	0,0%	
4	11	5,4%	1	0,5%	0	0,0%	
not applicable*****	2	1,0%	0	0,0%	0	0,0%	
**Cormack/Lehane** Score							
1	107	53,2%	4	2,0%	0	0,0%	0,1580** 0,0225***
2	21	10,4%	0	0,0%	0	0,0%	
3	0	0,0%	2	1,0%	0	0,0%	
4	0	0,0%	0	0,0%	0	0,0%	
not applicable*****	58	28,9%	7	3,5%	2	1,0%	
**Laryngoscopy method**							
Direct laryngoscopy	129	73,3%	6	3,4%	0	0,0%	0,0008****
Videolaryngoscopy	32	18,2%	7	4,0%	2	1,1%	

Abbreviations: *n* = number; %=percentage; BMI = body mass index; significance level = 0.05

* Kruskal-Wallis test; ** Spearman`s rank correlation coefficient; *** exact Cochrane-Armitage trend test (for analysing only patients with one or two intubation attempts); **** Fisher`s exact test (patients with fibreoptic intubation and awake tracheotomy are excluded); ***** Unavailable data

### Influence of the airway modality on the postoperative outcome

A difficult airway did not increase significantly the average intubation duration (*p* = 0,5992, Wilcoxon two-sample test) (Table 5). Similarly, no correlation between a difficult airway and a prolonged postoperative ICU stay and total hospitalisation length was detected (*p* = 0,8141/ *p* = 0,9007 respectively, Wilcoxon two-sample test) (Tables 6 and 7).

**Table 5:** Association between difficult airway and intubation duration

Difficult airway	Number of patients		Average length (days)	Range (days)		Standard-deviation	*p*-Value
	n	%		Min	Max		
yes	15	7,5%	0,67	0	1	0,49	0,5992*
no	186	92,5%	0,81	0	13	1,03	

Abbreviations: *n* = number; %=percentage; significance level = 0.05

* Wilcoxon Two-Sample Test

**Table 6:** Association between difficult airway and postoperative ICU stay

Difficult airway	Number of patients		Average length (days)	Range (days)		Standard-deviation	*p*-Value
	n	%		Min	Max		
yes	15	7,5%	0,73	0	2	0,59	0,8141*
no	186	92,5%	0,84	0	16	1,24	

Abbreviations: *n* = number; %=percentage; significance level = 0.05

* Wilcoxon Two-Sample Test

**Table 7:** Association between difficult airway and total postoperative hospitalisation length

Difficult airway	Number of patients		Average length (days)	Range (days)		Standard-deviation	*p*-Value
	n	%		Min	Max		
yes	15	7,5%	12,73	3	24	7,38	0,9007*
no	186	92,5%	12,49	1	35	6,70	

Abbreviations: *n* = number; %=percentage; significance level = 0.05

* Wilcoxon Two-Sample Test

136 patients underwent intraoperative tracheostomy. In these patients, the average intubation duration was 1,04 ± 1,07 days with a minimum of zero (intubation only for the duration of the day of surgery) and a maximum of 13 days respectively. The 65 patients without a tracheostomy had an average intubation duration of 0,29 ± 0,58 days with a minimum of zero (intubation only for the duration of the day of surgery) and a maximum of three days respectively. Patients with an intraoperative tracheostomy had a statistically significant longer intubation time than patients without tracheostomy (*p* < 0.0001, Wilcoxon two-sample test).

The 136 patients with an intraoperative tracheostomy had an average total postoperative hospitalisation length of 15,25 ± 5,78 days with a minimum of four and a maximum of 35 days respectively. The 65 patients without a tracheostomy had an average total postoperative hospitalisation length of 6,78 ± 4,69 days with a minimum of one and a maximum of 23 days respectively. Patients with an intraoperative tracheostomy had a statistically significantly longer total postoperative hospitalisation length than patients without tracheostomy (*p* < 0.0001, Wilcoxon two-sample test).

## Discussion

We aimed to specify the clinical features that predict a difficult airway and difficult intubation in 201 patients with surgical treatment of oral cavity carcinoma to enable future individualized health care in this patient cohort. Our results identified no patient-specific predictors for a difficult airway in the present study collective. A positive smoking and alcohol anamnesis, ASA score 2–3 and tumour T stage 2–3 were documented more frequent in the 15 patients with difficult airway; however, these variables cannot be generalized and should be interpreted only limited as key characteristics of this subgroup because of the very small sample.

No specific gender or age group was found to be at increased risk for a difficult airway in this study. Previous research suggests although that male patients may have an increased risk of a difficult airway, in terms of mask ventilation or laryngoscopy, however, in different cohorts with different disease patterns [14, 27–30]. Regarding the association of patient’s age and a difficult airway, the current literature is not clear. On the one hand, it appears to exist a fundamental association between increased age and difficult mask ventilation or laryngoscopy; on the other hand, there are indications that the risk of difficult laryngoscopy or intubation peaks in middle-aged patients and appears to decrease again with increasing age [14, 15, 27, 28, 31–33].

An increased BMI was not associated with increased incidence of difficult airway in this study. Due to the various definitions of difficult mask ventilation and difficult intubation in the literature, a valid comparison with the present results is difficult, even if considering the sub-definitions of difficult airway [11, 15, 28, 29, 32, 34]. Although Kheterpal et al. were unable to find an association between an increased BMI and impossible mask ventilation, their results aren’t comparable to ours, as no difficult mask ventilation was reported [28]. Moon et al. found no significant association between their two defined BMI groups and difficult intubation, which is consistent with the present results [33]. However, other studies could demonstrate an association between an increased BMI and difficult intubation [15, 29, 34]. In the study of Saasouh et al. more than one intubation attempts were required in 9% of the cases, which is similar to 7,5% in our study [12]. When analysing the entire patient population, Saasouh et al. found that an increasing BMI up to 30 was correlated with increased risk for difficult intubation; however, no correlation was found in patients with BMI ≥ 30 [12]. Due to the definition’s differences of difficult intubation between studies, no comparison was possible with the S1 guideline from DGAI [15, 29, 34].

Regarding the intraoral tumour localisation, we hypothesized that a more dorsally located tumour intraorally, like at the posterior floor of the mouth, could lead to a difficult laryngoscopy through the potential restriction of the glottis view. The study results did not confirm this hypothesis. A comparable study by Akadiri et al. with 28 patients receiving surgical treatment of oral cavity carcinomas could also not identify a specific tumour localisation predictive for a difficult intubation [35]. However, we can assume that a tumour localisation that could restrict the glottis view would lead anaesthesiologists to use an alternative laryngoscopy method (e.g. videolaryngoscopy) for intubation in advance, reducing the risk of a potential difficult airway compared to direct laryngoscopy [36].

Regarding the tumour stage, we hypothesized that incidence of difficult airway would be increased with an advanced T stage. However, none of the documented tumour stages was found to be associated with increased incidence of difficult airway. Nine out of 15 patients (60%) with difficult airway had a tumour T stage 2–3; however, without statistical significance. No similar studies exist to date in the international literature to compare with our results. Still, no generalized conclusion can be derived only from our study collective. It can be thought that an advanced tumour stage may prompt anaesthesiologists to use an alternative laryngoscopy method in advance. Further prospective studies should evaluate if this could reduce the risk of a difficult airway, possibly explaining the present findings [36].

In a recent meta-analysis by Hung et al. pre-irradiation of the neck was identified as a risk factor for difficult mask ventilation [37]. This contrasts with the present results, in which none of the pre-irradiated patients had a difficult airway, and those of Sharma et al. and Zhang et al. [7, 38]. Sharma et al. compared the incidence of difficult intubation in patients who underwent head and neck tumour surgery after neoadjuvant radiotherapy with the incidence of difficult intubation in previous surgeries of the same collective and found no statistical association [7]. According to our clinical experience, pre-irradiation leads to significant constriction of the intraoral and cervical soft tissues, especially in revision surgeries, and airway management could be challenging. Thus, our results need to be validated by future studies.

According to the literature, an increased ASA score, could increase the risk of a difficult airway in surgeries under general anaesthesia [14, 15, 31]. However, a comparison with the present study is difficult due to the different patient population, as this study refers to patients with oral cavity cancer exclusively. However, Schnittker et al. documented the most frequent difficult airways in patients with ASA scores 1 and 2, compared patients with ASA score 3 in the present study [15]. Even if 94% of our study patients with a difficult airway presented an ASA score of 2–3, no generalized conclusions can be derived due to the small sample of this subgroup.

Of the 22 patients who underwent revision surgery, 12 were intubated using direct laryngoscopy and 9 using a videolaryngoscope. One patient received an awake tracheostomy under local anaesthesia after preoperative interdisciplinary evaluation of the definitive compromised airway. Local anatomical alterations of the intraoral and extraoral soft tissues after primary surgery may lead to trismus, that could potentially affect the airway management. Consequently, anaesthesiologists may anticipate a more difficult airway management during revision surgery [6, 16]. A difficult airway was reported in none of the patients with revision surgery. We assume that anaesthesia providers would certainly have been aware of the re-surgery in advance. The careful preoperative anaesthesiological approach in this cohort and decision for use of videolaryngoscope in almost half of the cases in advance may explain this finding. This hypothesis is also supported by Mishra et al., after all their study patients with restricted mouth opening or revision surgery for oral cavity carcinoma received fibreoptic intubation in advance [39]. Moreover, recent randomized controlled trials are highlighting the use of indirect laryngoscopy (e.g. videolaryngoscopy) for intubation, which can result in a significantly higher incidence of a successful first intubation attempt [40–42].

Bhatnagar et al. concluded that an increased Mallampati score alone has poor predictive power for the incidence of a difficult laryngoscopy but postulated no significant correlation [43]. In the meta-analysis of Shiga et al., in which patients without airway pathologies were examined, 5.7% of patients with a Mallampati score of ≥ 3 had a difficult intubation [44]. Our study reported an incidence of 0.5%. This difference could indicate that anaesthesiologists are more likely to anticipate the possibility of a difficult airway in patients with oral cavity cancer and may therefore choose an alternative laryngoscopy method in advance. An increased Mallampati score and a reduced mouth opening or head reclination may also influence each other and be associated with each other, showing the multifactorial nature of the cause for a difficult airway [45]. This would further suggest that alternative indirect laryngoscopy methods should be used more frequently in patients with oral cavity carcinoma, as in the present study. In this case, the incidence of a difficult airway would probably be lower than with direct laryngoscopy [36].

Previous research of Wilson et al. and Karkouti et al. found a significant correlation between restricted mouth opening and difficult intubation [46, 47]. These results are underlined by a prospective double-blind study of Chhina et al. [48]. This contrasts with the meta-analysis of Shiga et al. in which the authors were unable to clearly identify restricted mouth opening as a risk factor for difficult intubation [44]. However, they argue that a restricted mouth opening may be challenging for a direct laryngoscopy and conclude recommending further investigations into this correlation due to possible bias [44]. The present authors suggest an alternative intubation method (e.g. videolaryngoscopy) for the first intubation attempt in patients with restricted mouth opening. Similarly, Wilson et al. and Karkouti et al. also found a significant association between restricted head reclination and difficult intubation [46, 47]. This is also underlined by Chhina et al. [48]. They also postulated that restricted head reclination in combination with other tests (e.g. Mallampati score and neck circumference) may have a high sensitivity in predicting difficult intubation [48]. A direct comparison with the present results is difficult, due to differences in the definition of difficult laryngoscopy and intubation [46–48].

Regarding the number of intubation attempts needed, Lundstrom et al. stated Mallampati score of ≥ 3 as a significant risk factor for difficult intubation with increased number of intubation attempts, in addition to other factors [34]. Identical results were reported in the study of Moon et al. in patients with a Mallampati score ≥ 3 in addition to other factors [33]. In the present study, an alternative indirect laryngoscopy method was preferred in 33% of patients, which could explain the lower number of patients who required increased intubation attempts and why there was no significant association between an increased Mallampati score and an increased number of intubation attempts. The present results showed that a Cormack/Lehane score of 3 had a higher association with two intubation attempts required than patients with a Cormack/Lehane score of < 3. Our reported difficult intubation of 7.5% was similar to the results of Bilgin et al. and slightly lower than Dawood et al. who documented and incidence of 8% [49, 50]. Both studies investigated screening tests for difficult intubation in a patient population with general surgery. Both these studies and our results confirm the theory of Cormack and Lehane that a score of 3 could difficult intubation performed by direct laryngoscopy [13]. Unfortunately, the data quality of this study doesn’t allow us to derive which attempt was performed with direct or video laryngoscopy.

Unlike what we hypothesized, the success rate of first intubation attempt was with 78% significantly lower with videolaryngoscopy, compared to 95,5% when direct laryngoscopy was used. Accordingly, the rate of difficult intubation with videolaryngoscopy was significantly higher than with direct laryngoscopy (21,9% vs. 4,4%). These findings contradict the results of current international randomized controlled trials, which reported higher first-pass success when using videolaryngoscope [40–42]. A lower training status of the anaesthesia providers or an insufficient anaesthesiological preparation could explain the lower first-pass success in the videolaryngoscopy group in our study. However, we believe that our anaesthesiologists decided for using videolaryngoscopy in advance in these 41 study patients after a very careful preoperative assessment with clearly defined criteria for a difficult airway. But even then, a higher success of the first intubation attempt was not guaranteed, like our results showed. The authors although support the suggestion of current international randomized controlled trials for the use of videolaryngoscopy as a preferable approach for a successful first intubation attempt, especially in patients undergoing revision surgeries [40–42]. However, these studies are referring to patients receiving general surgical procedures and not specifically surgeries of oral cavity carcinoma. Further prospective studies documenting the exact laryngoscopy approach at each intubation attempt would be useful in clinical practise for this patient population.

The hypothesis of this study was that the occurrence of a difficult airway prolongs the intubation time, the postoperative time in the intensive care unit and the postoperative hospital stay of affected patients. At the time this study was written, no other study had examined the postoperative time course of patients with oral cavity carcinoma. Our data refuted our initial hypothesis and suggested that a difficult airway does not appear to prolong the postoperative course. However, it should be mentioned here that the duration of the postoperative course may be multifactorial and that other factors beyond those investigated in this study may also have an influence on its duration [51–54].

Regarding the duration of intubation, the hypothesis that intraoperatively tracheotomised patients have a longer intubation time than non-tracheotomized patients was confirmed in our study. Nagarkar et al. in their study of patients with surgical treatment of head and neck tumours reported a tracheostomy rate of 2,6% [23]. An alternative laryngoscopy method (fibreoptic) was used in 69,2% of the cases, higher than the 33% in this study [23]. Nagarkar et al. postulated that 75,4% of 500 patients were extubated within six to eight hours and 24.6% at 14 h postoperatively [23]. If 14 h postoperatively was the morning of the first postoperative day, this rate is lower than 68,7% of the present study. This difference could be explained by possible differences in the standard postoperative procedure. No direct comparison between the duration of intubation of tracheotomised and non-tracheotomised patients with surgical treatment of oral cavity carcinoma can be found in the literature. Although an intraoperative tracheostomy can significantly prolong the intubation time, this cannot be generalized because the intubation time is multifactorial and depends on various individual surgical, anatomical and anaesthesiological variables [51–54].

In the present study, tracheostomised patients had a significantly longer postoperative stay (15.3 days on average) than non-tracheostomized patients (6.8 days on average). This is consistent with Nagarkar et al. who reported 7.6 and 4.2 days for their patient cohort respectively [23]. The difference in the absolute values of the length of stay in the two studies could be explained by the fact that in the study by Nagarkar et al. the rate of tracheotomised patients was significantly lower (2,6%) compared to our study (67,7%) [23]. The results of Myatra et al. (11.5 and 7.2 days respectively) are also consistent with the present study [21]. However, Myatra et al. also found a significantly higher rate of postoperative complications, both surgical and airway-related, in the group of tracheostomised patients [21]. Tumour-specific factors like an increased T stage mostly result to extended reconstructive surgery. Since intraoral tumour resection followed by free flap reconstruction, especially when combined with bilateral neck dissection, can often lead to significant postoperative cervical swelling or haemorrhage, we perform tracheotomy to reduce the postoperative risk of compromised airway in this patient cohort. Additionally, tracheotomized patients often need a gradual process to wean off mechanical ventilation or supplemental oxygen and also require physical, speech, and swallowing training as part of their recovery. This necessity extents in cases of concomitant severe medical conditions, such as chronic obstructive pulmonary disease. Due to these factors, tracheotomized patients can necessitate extended care and rehabilitation in a hospital setting compared to those who do not underwent the procedure, fact that was confirmed by our study results.

There are some limitations to the current study. The retrospective nature of this observational research could lead to documentation bias. Especially the data of which laryngoscopy method was used by each intubation attempt were not available sufficiently, e.g. it was not always clearly documented which laryngoscopy method was used for further intubation attempts after the first unsuccessful attempt using direct laryngoscopy. Thus, a safe conclusion for clinical practise cannot be obtained. Although our patient collective was similar to relevant studies with patients with oral cavity carcinomas, our sample of 201 patients could be insufficient for a valid conclusion [39, 55]. Further multicentre studies with a clearly larger collective are needed in order to extract safer results. Second, the attending anaesthesiologists had different levels of training and experience. Consequently, observer bias may have occurred since medical records were completed by the responsible and thus, the difficulty of airway management may have been assessed exclusively subjectively, which limits the generalisability of our results. Third, retrospectively it is not possible to understand why an anaesthesiologist chose an alternative indirect laryngoscopy method in the first place, bias that could mask an even higher incidence of difficult airway. Fourth, further predictive factors for a difficult airway, such as a restricted mandibular protrusion, edentulism or sleep apnoea syndrome, could not be extracted from the medical records. Fifth, the collective inhomogeneity due to the small number of patients in the subgroups of tumour localisation may also lead to bias by underestimating this as a real risk factor for a difficult airway. As we still believe that tumours of the mouth of floor and posterior tongue do associate with a difficult airway, further studies with a larger collective would be of great clinical interest. Sixth, the influence of a difficult airway and an intraoperative tracheostomy on the postoperative course could bias the results interpretation, as the postoperative course is not exclusively influenced only by these two factors.

This study is unique in examining a collective with oral cavity carcinoma treated with an interdisciplinary approach with reference to the S1 guideline of the DGAI and provides valuable information specifically for the national health care, but also in general, as this guideline is similar in principle to those of the UK Difficult Airway Society 2015 guidelines.

## Conclusions

Within the limitations of this study, no patient-specific predictors for a difficult airway were identified in patients with oral cavity carcinoma. Videolaryngoscopy in advance did not increase the success rate of the first intubation attempt compared to direct laryngoscopy. Despite this, videolaryngoscopy may be a preferable approach in this population, especially in patients undergoing revision surgeries. The results highlight the importance of a careful preoperative assessment with clearly defined criteria for a difficult airway and appropriate anaesthesiological preparation to avoid complications during intubation.

## Data Availability

The datasets used and/or analysed during the current study available from the corresponding author on reasonable request.

## Declarations

### Presentation

Preliminary data for this study were presented as a poster presentation at the 27th EACMFS Congress, 17–20 September 2024, Rome.
